# A novel antimicrobial peptide, Ranatuerin-2PLx, showing therapeutic potential in inhibiting proliferation of cancer cells

**DOI:** 10.1042/BSR20180710

**Published:** 2018-11-09

**Authors:** Xiaoling Chen, Luyao Zhang, Chengbang Ma, Yingqi Zhang, Xinping Xi, Lei Wang, Mei Zhou, James F. Burrows, Tianbao Chen

**Affiliations:** 1 School of Pharmacy, Queen’s University Belfast, Belfast BT9 7BL, Northern Ireland, U.K.; 2 Department of Emergency Medicine, The First Hospital of Hebei Medical University, Shijiazhuang 050031, China

**Keywords:** anuran skin secretions, antibacterial properties, anti-cancer, peptides, rana-box

## Abstract

Antimicrobial peptides are a promising resource for developing novel antibiotic and even anticancer drugs. Here, a 28-mer polypeptide, Ranatuerin-2PLx (R2PLx), was identified from lyophilised skin secretions. The chemically synthetic replicates exhibited moderate and broadspectrum antimicrobial effect against various microorganisms including methicillin-resistant *Staphylococcus aureus* (MRSA, minimal inhibitory concentration = 256 µM). In addition, R2PLx was found to inhibit the proliferation of several tumour cells, especially showing more potent effect on prostate cancer cell, PC-3. The early cell apoptosis was observed in 6 h by Annexin V-FITC/propidium iodide staining, as well as the activation of Caspase-3 at 5 µM peptide concentration. R2PLx may therefore be promising for developing new therapeutic approach for cancer treatment. Moreover, the artificial deficiency of conserved rana-box loop or net positive charge in C-terminal domain notably reduced the biological activities of the truncated and substituted isoforms, respectively, suggesting for maintaining their biological potency of ranatuerin family requires both cysteine-bridged segment and cationincity within the loop domain in C-terminus.

## Introduction

As a crucial part of host innate defensive system, antimicrobial peptides (AMPs), secreted by amphibian granular glands, are ideal candidates to examine as potential novel antibiotic alternatives which could combat multidrug resistant bacterial strains [1–3]. Apart from their broadspectrum antibacterial capabilities, reports have also indicated that these peptides may also have potential in other fields due to their tumouricidal, antiviral and immunomodulatory properties [4]. Previous research has revealed these amphibian skin-derived AMPs are diverse in their primary sequences, though they are commonly hydrophobic, cationic and able to form an amphipathic helix in a membrane-mimetic solvent [4,5].

Ranatuerins, a canonical family of anuran AMPs, was initially identified from genus *Rana* and generally they display broadspectrum antibacterial activity against a range of ubiquitous pathogens, along with comparatively low haemolytic activity [6–8]. Ranatuerin-2 is a predominant family member which consists of a N-terminal α-helix domain and contains the conserved domains, including Gly^1^, Lys^22^ and the heptapeptide C-terminal ‘rana box’ [8–11]. Several similar isoforms have also been identified from North American *Rana* frogs, such as ranatuerin-2VEa and -2VEb, and ranatuerin-2Oa and -2Oe have been identified from the Chinese bamboo leaf odorous frog and Japanese brown frogs [9,10].

Herein, we describe the discovery of a biosynthetic precursor, preproranatuerin-2, encoding an antimicrobial peptide, Ranatuerin-2PLx (R2PLx), from the skin secretions of the pickerel frog (*Rana palustris*) using a combination of ‘shotgun’ cloning and mass spectrometry. The corresponding chemically synthesised replicate exerted antibacterial activities towards pathogenic microorganisms and antiproliferative activity towards tumour cells by inducing apoptosis. In addition, we demonstrate that deleting the conserved rana-box loop drastically reduces the antibacterial and antiproliferative activities of this peptide.

## Materials and methods

### Acquisition of dermal secretions from pickerel frog

Twelve specimens of mature pickerel frog (scale between 4 and 5 cm length) were exposed to 12 h of light at 18–25°C daily and multivitamin loaded crickets were provided as the fodder threetimes/weeks. Following 4-month breeding, dermal secretions were obtained via surface electrical stimulation introduced by Tyler and co-workers in 1992 under proper Home Office (U.K.) animal licences (project licence PPL 2694). In summary, the skin surface was moistened with deionised water, followed by two periods of transdermal electric stimulation (6 V, 50Hz, 4ms pulse width), each of 20 s duration. Finally, the secretions collected by gently flushing the frog skin with deionised water were lyophilised and stored at −20°C until mRNA extraction.

### Cloning and sequencing of a cDNA encoding R2PLx peptide precursor

The intact polyadenylated mRNA was refined from crude lysate among 5 mg of lyophilised secretions via Dynabeads^®^ mRNA DIRECT™ Kit (Invitrogen). Following reverse transcription, the full-length sequence of the mRNA transcript encoding the R2PLx peptide precursor was captured using the SMART-RACE kit (Clontech U.K.). Specifically, a supplied NUP primer and a degenerate primer (S1; 5′-GAWYYAYYHRAGCCYAAADATG-3′) were employed in molecular cloning. In addition, the degenerate primer was designed to a highly conserved domain of the 5′-UTR of previously characterised homologous peptide cDNAs from *Rana*. Thereafter, RACE-products were subjected to purification, molecular cloning and sequenced using Cycle Pure Kit (Omega Bio-Tek, U.S.A.), pGEM-T vector system (Promega Corporation) and ABI 3100 automated sequencer respectively.

### Elucidation of R2PLx in structural characterisation

After centrifugation (2500×***g***, 5 min) from 10 mg/ml secretion solution dissolved in 0.05/99.5 (v/v) trifluoroacetic acid (TFA)/water, the clear supernatant was subjected to RP-HPLC column (Jupiter C5; 250 mm × 4.6 mm, Phenomenex, U.K.) on a Cecil CE4200 Adept gradient system (Cambridge, U.K.) (a gradient formed from 0.05/99.5 (v/v) TFA/water to 0.05/19.95/80.0 (v/v/v) TFA/water/acetonitrile in 240 min at a flowrate of 1 ml/min). Subsequently, fractions were taken every minute and each was analysed using MALDI-TOF mass spectrometer (α-cyano-4-hydroxycinnamic acid as the matrix) on a linear time-of-flight Voyager DE mass spectrometer (Perseptive Biosystems, MA, U.S.A.). The fractions with masses coincident with putative novel cDNA-encoded peptide were subjected to primary structural analysis by MS/MS fragmentation sequencing using an LCQ-Fleet electrospray ion-trap mass spectrometer (Thermo Fisher Scientific).

### R2PLx and related analogues synthesis and purification

To investigate the effect of the ring structure, the antimicrobial peptide R2PLx was used as the framework to design a deletion analogue where the C-terminal hexapeptide ring was removed, namely R2PLx-22. In addition, for better understanding the dominant influence of loop domain, lysine residues at position 24 was replaced with serine to eliminate the net positive charge within the loop structure, namely S^−24^-R2Plx (GIMDTVKNAAKNLAGQLLDKLKCSITAC). Sufficient quantities to evaluate the bioactivities of each peptide was obtained using the automatic PS4 peptide synthesizer (Protein Technologies, U.S.A.) along with standard Fmoc-chemistry. Cleavage of the primary products from the resin and subsequent deprotection used a mixture of TFA, ethanedithiol, thioanisole and water (94:2:2:2 [v/v]). Prior to lyophilisation, R2PLx and S^−24^-R2Plx were dissolved in 0.2% of hydrogen peroxide in 0.05/19.95/80.00 (v/v/v) TFA/ water/ acetonitrile for 30 min to get the loop structure via sulfhydryl oxidisation. Finally, each synthetic peptide was purified using RP-HPLC (Phenomenex C-5 column, 0.46 × 25 cm), and its purity was confirmed using MALDI-TOF mass spectrometry (Perseptive Biosystems, MA, U.S.A.).

### Circular Dichroism spectra

The secondary structure of R2PLx and C-terminal deleted analogue were estimated using a circular dichroism (CD) spectrometer (Jasco J851, U.S.A.). Specifically, the parameters were 100 µM of each peptide, which was dissolved in 10 mM ammonium acetate (NH_4_AC) buffer or 50% trifluoroehanol (TFE) (v/v in 10 mM NH4AC) respectively, loaded in a cuvette (1-mm path length). For the analysis, three passes (‘accumulation’) within the range of 190–260 nm were made at 20°C at a scanning speed of 200 nm/min, a bandwidth of 1 nm, and a data pitch of 0.5 nm. The percentage of the α-helix structure was predicted via K2D method in DichroWeb website (http://dichroweb.cryst.bbk.ac.uk/html/home.shtml).

### Antimicrobial assay

The minimal inhibitory concentration (MIC) of both synthesised peptides were determined against *Staphylococcus aureus* (NCTC 10788), *Escherichia coli* (NCTC 10418) and *Candida albicans* (NCTC 1467) as well as the resistant microorganisms MRSA (ATCC 12493), *Enterococcus faecalis* (NCTC 12697) and *Pseudomonas aeruginosa* (ATCC 27853), each of which had been cultured in Mueller–Hinton broth. Cultures of each microorganism (1 × 10^5^ colony forming units (CFU)/ml) were inoculated with peptide solutions in a concentration range of 1–512 μM (in 2-fold dilutions) in a 96-well plate (100 μl per well) and incubated at 37°C in a humidified atmosphere for 16–24 h. Thereafter, the absorbance values of each well was determined at 550 nm using a Synergy HT plate reader (Biotech, U.S.A.) and the MIC was defined as the lowest concentration of the respective peptide that resulted in no apparent growth of the microorganism. In addition, 20 μl of a mixture from each well was inoculated on Mueller–Hinton agar plates. The corresponding peptide concentration where no bacterial communities grew was defined as the minimum bactericidal concentration (MBC).

### MTT cell viability assay and Lactate dehydrogenase leakage assay

Each of the five cancer cell lines — non-small cell lung cancer H157, melanocyte MDA-MB-435S, human prostate carcinoma PC-3, human glioblastoma astrocytoma U251MG, human breast cancer MCF-7 — as well as the cell line for normal human microvessel endothelial cells HMEC-1 were seeded into a 96-well plate at densities of 5000 cells/well. After incubation for 24 h at 37 °C with 5% CO_2_, the cells were starved for 6 h by replacing the medium with serum-free medium. Thereafter, synthesised peptides (in 10-fold concentrations from 10^−4^ to 10^−9^ M in serum-free medium) were incubated with the cells for 24 h after which 10 μl of MTT solution (5 mg/ml) was added to each well under dark conditions. Following a further 4 h of incubation, 100 μl of DMSO superseded medium was replaced to each well to dissolve the formazan crystals. The OD value of each well was read by the Synergy HT plate reader at 550 nm.

The cell (PC-3 cells) loss of intracellular LDH was measured via LDH Cytotoxicity Assay Kit (Thermo Fisher Scientific). Equivalent cells treated with sterile water or lysis buffer (supplied with the kit) for 45 min served as the spontaneous and maximum LDH activity controls, respectively. Then, 50 μl of supernatant was collected from each well and mixed with equal volume of supplied reaction mixture and incubated at room temperature for 30 min. Fifty microlitre of supplied stop solution was added to each well. The absorbance at 680 nm was measured using Synergy HT plate reader and subtracted from the 490 nm absorbance. Similarly, to obtain the kinetics of cytoplasmic LDH release, 50 μl of supernatant from equivalent PC-3 cells treated with 100 μM, 10 μM and 1 μM of R2PLx peptide, respectively, at different time points (6, 12, 24 and 36 h), was collected and the changes in the release of cytoplasmic LDH were monitored.

### Annexin V-FITC/ propidium iodide staining assay

The fluorescence monitoring of cellular death was evaluated through FITC Annexin V Apoptosis Detection Kit (BD Bioscience), based on the published method [12]. Briefly, PC-3 cells were seeded into 24-well plate (3 × 10^4^ cells/well). After 24 h of incubation and 6 h of serum starvation, the cells were treated with 5 μM of R2PLx. At different time points (0, 6 and 24 h), cells were supplemented with 400 μl of binding buffer containing 5 μl of Annexin V-FITC and 5 μl of propidium iodide (PI), and then were incubated for 10–15 min at room temperature in the dark. Thereafter, analysed in images were captured by fluorescence microscopy (Leica Microsystems, Switzerland).

### Detection of Caspase-3 activity on PC-3 cells

Activation of Caspase-3 in R2PLx induced prostate cancer cells was measured using EnzChek Caspase-3 Assay Kit (Molecular Probes, U.S.A.), according to the manufacturer’s instructions.

Briefly, after 24 h of incubation and 6 h of serum starvation as described previously, 1 × 10^6^ cells of PC-3 cells was treated with 5 μM of R2PLx for 6 h. Equivalent cells treated with serum-free medium served as control group. Each cell sample was resuspended in 50 μl of the 1× cell lysis buffer (supplied with the kit) and set on ice for 30 min. Thereafter, 50 μl of each supernatant (centrifuged at 2500×***g*** for 5 min at 4°C) was mixed thoroughly with an equal volume of 2× reaction buffer (supplied with the kit) and incubated for 1.5 h. In addition, 50 μl of the 1× cell lysis buffer was taken as the background fluorescence of the substrate. The fluorescence intensity was measured (excitation at 360 nm and emission at 460 nm) using an ELISA plate reader (Biolise BioTek EL808).

### Haemolysis assay

To determine the toxicity of the peptides to normal mammalian cells, a 2% solution of horse erythrocytes was resuspended in PBS solution and incubated with each peptide at the standard concentrations (i.e., from 1 to 512 μM) for 2 h at 37 °C. Equivalent cells treated with PBS or 1% Triton X-100 served as the positive and negative controls, respectively. Following centrifugation at 900×***g*** for 5 min, 100 μl of the supernatant from each tube was transferred to a 96-well plate, which was then read at 550 nm using the Synergy HT plate reader.

### Statistical analyses

Statistical analyses of all bioactivity assays were performed using Prism 6 (GraphPad Software, U.S.A.). Data points represent the average of three independent experiments with error bars presenting the S.E.M.

## Results

### Molecular cloning of biosynthetic precursor encoding cDNA and structural characterisation of R2PLx peptide from RP-HPLC fraction of skin secretion

**Figure 1:**
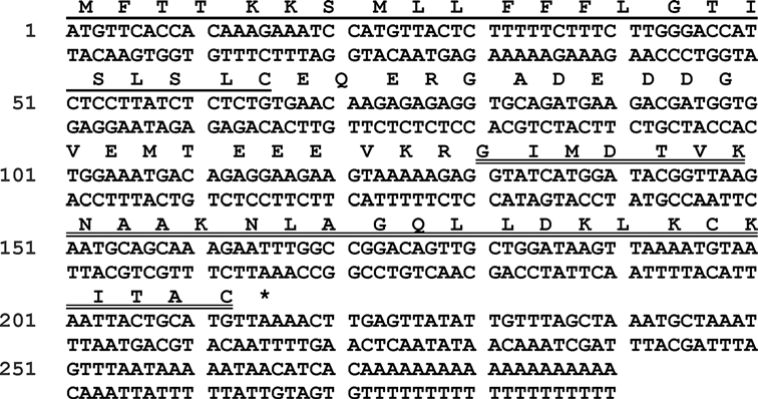
Nucleotide sequence and corresponding translated ORF of precursor cDNA cloned from the pickerel frog (*R. palustris*) tegmental secretion library which encodes R2PLx peptide The putative signal peptide and mature peptide are labelled by single-underline and double-underline respectively. The asterisk corresponds to the stop codon. The cDNA sequence has been deposited in the NCBI Database (accession code; MG872823).

**Figure 2:**
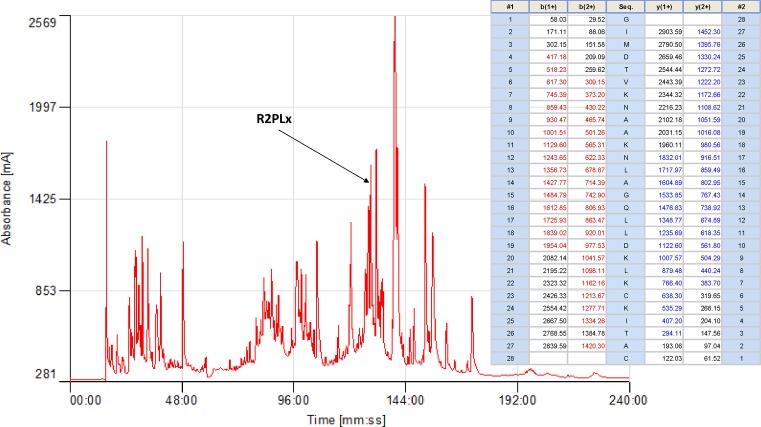
Full region of RP-HPLC chromatogram of pickerel frog (*R. palustris*) skin secretion The retention time of R2PLx is shown by the arrow. The masses of corresponding b and y ions derived from R2PLx in the table are indicated in red and blue, respectively.

The nucleotide sequence of a full-length biosynthetic precursor encoding cDNA was consistently cloned among the secretion-derived cDNA library (Figure 1), compromising 71 amino acid residues. From the translated open reading frame (ORF), the typical signal peptide consisting of 22 amino acid residues was determined through NCBI-BLAST. The typical convertase processing site, -KR-, indicates the start of the mature peptide. The mature 28-mer peptide, which we have named R2PLx, also appears to have a C-terminus which is subjected to post-translational modification with a disulphide cysteine-loop (present in and accounted for by molecular mass in MS afterwards). The R2PLx biosynthetic precursor encoding cDNA has been deposited in the NCBI Database (accession code; MG872823) and the presence of the mature R2PLx peptide in the secretions was further confirmed by RP-HPLC isolation and MS/MS fragmentation sequencing, with the retention times at 128 min (Figure 2).

### Synthesis and secondary structure analysis

**Figure 3:**
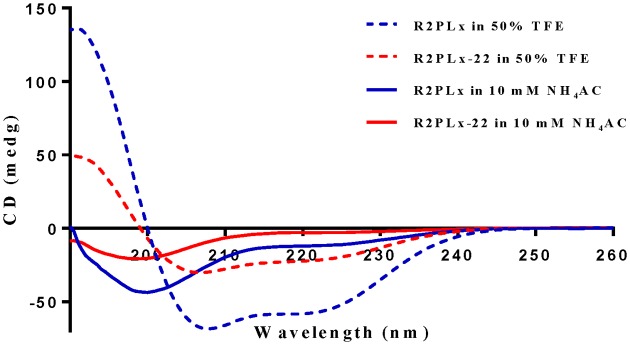
CD spectra of R2PLx (Blue) and R2PLx-22 (Red) in different solutions The CD spectra in 50% TFE/10 mM NH4AC solution and 10 mM NH4AC buffer at 20°C are indicated by dotted and solid line, respectively.

Although ranatuerins possess the ‘rana box’ domain at their C-terminii which is also present in other peptide families from the brevinin superfamily, the structural characteristics, especially the amino acid constitution of the ‘rana box’, are variable. Therefore, to further investigate the influence of the ‘rana box’ of R2PLx on its biological activity, both R2PLx and the ‘rana box’ deleted analogue, R2PLx-22, were synthesised and purified (Table 1). The CD spectra of both peptides shows the pattern of random coil in aqueous solution and a typical α-helix CD spectra in 50% of TFE solution (Figure 3). Obviously, R2PLx shows a more negative peak at 222 and 208 nm than R2PLx-22, indicating larger α-helical content formed in the membrane-mimetic environment. The calculation of helicity of R2PLx is nearly 2-fold then which of R2PLx-22 (Table 1).

**Table 1:** The amino acid sequences and the calculated α-helicity in 50% of TFE solution of R2PLx and the analogue

Peptide	Sequence	α-helix in 50% TFE (%)
**R2PLx**	GIMDTVKNAAKNLAGQLLDKLKCKITAC	78
**R2PLx-22**	GIMDTVKNAAKNLAGQLLDKLK	40

### Antimicrobial activity

R2PLx and its two analogues have antimicrobial activity against five of the six microorganisms tested (Table 2). R2PLx shows higher potency than the truncated analogue on all the tested microorganisms except *P. aeruginosa*. R2PLx also exhibits antimicrobial activity towards MRSA although this activity is less potent than that towards the wild-type *S. aureus*. These results would indicate the removal of the ‘rana box’ markedly decreases the antimicrobial activity of the parent peptide. Surprisingly, the deficiency of cationic amino acid inside such motif, greatest impairs the antibacterial activity of this rana-box AMP.

**Table 2:** Antimicrobial activity of R2PLx peptide and its analogues against various microorganisms

Microorganisms	MIC/MBC (µM)		
	R2PLx	R2PLx-22	S^−24^-R2PLx
*S. aureus* (NCTC 10788)	32/128	256/256	>512/>512
*E. coli* (NCTC 10418)	32/64	128/256	512/>512
*C. albicans* (NCTC 1467)	256/512	512/>512	>512/>512
MRSA (ATCC 12493)	256/512	>512/>512	>512/>512
*E. fecalis* (NCTC 12697)	128/512	>512/>512	>512/>512
*P. aeruginosa* (ATCC 27853)	>512/>512	>512/>512	>512/>512

### Antiproliferative activity

**Figure 4:**
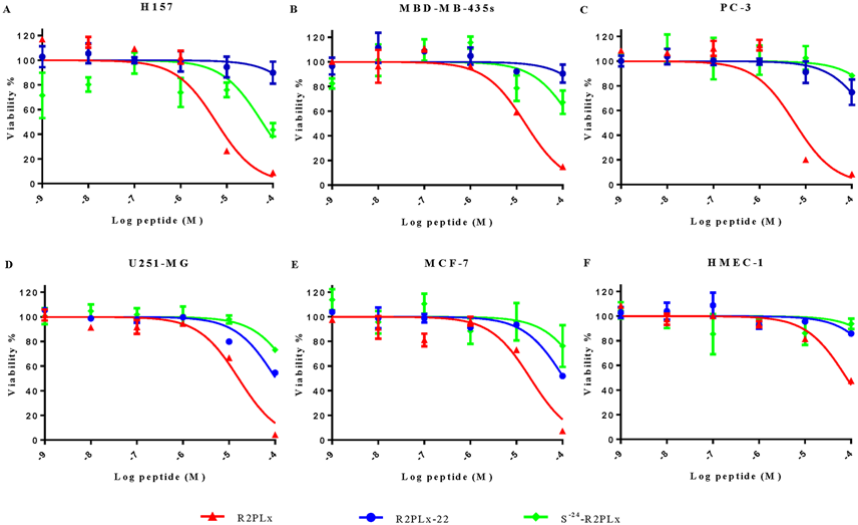
The cell viability of selected cell lines when treated with various concentrations of R2PLx (red), R2PLx-22 (blue) and S-24-R2PLx (green) (**A**) H157, non-small cell lung cancer, (**B**) MBD-MB-435, melanocyte, (**C**) PC-3, human prostate carcinoma cell line, (**D**) U251MG, human glioblastoma astrocytoma, (**E**) MCF-7, human breast cancer cell line and (**F**) HMEC-1, normal human microvessel endothelial cells. The curves were fitted using normalised dose-response algorithm. The error bar represents the S.E.M. of nine replicates, and the statistical significance was verified using one-way ANOVA, and it is illustrated in Supplementary Table S1.

R2PLx exhibits a dose-dependent inhibitory effect on the proliferation of the five tested human cancer cell lines (Figure 4). It inhibited the proliferation of these cell lines with IC_50_s ranging from 5.79 to 20.19 µM (Table 3). R2PLx-22 and S^−24^-R2PLx did show some activity against these cancer cells, but both were much less potent than R2PLx, indicating the alteration in loop domain of the C-terminus dramatically reduces its antiproliferative activity. The impact of R2PLx on the normal cell line HMEC-1 was also examined, and while it did have an impact upon the proliferation of these cells, it was much less potent towards these cells (IC_50_ 79.50 µM) than it was towards the cancer cells.

**Table 3:** The calculated IC50s of R2PLx and its two analogues were calculated from the normalised curves in Figure 4

Human cell lines	IC_50_ (μM)		
	R2PLx	R2PLx-22	S^−24^-R2PLx
H157	5.90	843.40	58.18
MBD-MB-435s	15.44	872.70	179.00
PC-3	5.79	283.90	792.60
U251-MG	16.14	104.10	278.30
MCF-7	20.19	109.30	316.90
HMEC-1	79.50	588.20	1185.00

### Haemolysis activity

**Figure 5:**
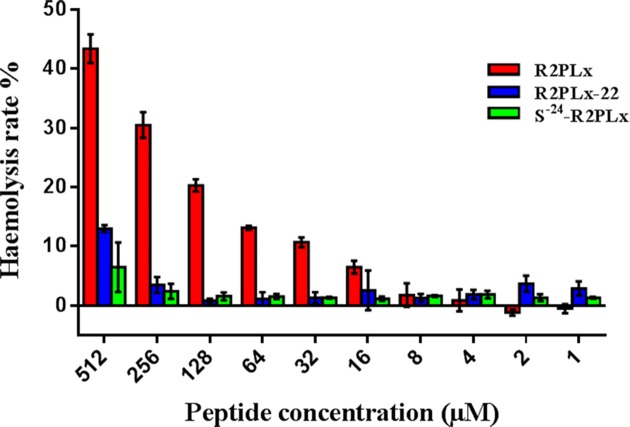
Haemolytic activity of R2PLx (red), R2PLx-22 (blue) and S-24-R2PLx (green) against the horse erythrocytes, presented by solid and striping columns, respectively The percentage is obtained by the comparison of haemolysis induced by 1% Triton X-100. The error bar represents the S.E.M. of three replicates.

R2PLx was found to possess moderate haemolytic activity in regard to horse erythrocytes, with less than 50% haemolysis at the highest concentration used in this assay. Indeed, the haemolytic activity of R2PLx was less than 5% at the IC_50_s against H157 and PC-3 cells, and less than 15% at the MICs for *E. coli* and *S. aureus*. Both analogues resulted in less haemolysis, even at higher concentrations, while the substituted analogue demonstrates minimal toxicity towards healthy mammalian cells (Figures 4 and 5).

### R2PLx-induced apoptosis on PC-3 cancer cell

**Figure 6:**
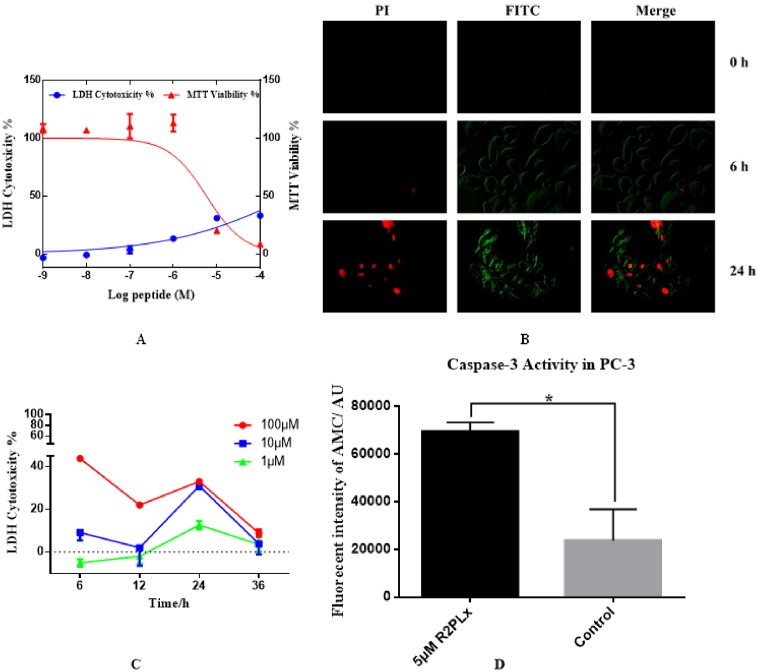
Detection of R2PLx on induction of cell apoptosis in PC-3 cells (**A**) The effect of R2PLx on PC-3 cells was estimated on both LDH (blue) and MTT (red) assays. The percentage of LDH cytotoxicity and MTT viability were obtained by compared with positive group (treated with lysis buffer) and growth control group (treated with medium). The error bar represents the S.E.M. of three replicates. (**B**) Annexin V-FITC/PI staining of PC-3 cells were evaluated after treating with R2PLx peptide at the IC50 value over different time scales. (**C**) Cytoplasmic LDH release of PC-3 cells treated with 100 µM (red), 10 µM (blue) and 1 µM (green) of R2PLx at 6, 12, 24 and 36 h respectively. (**D**) Caspase-3 activity on PC-3 cells after 6 h of incubation with 5 µM R2PLx (black) with respect to untreated cells (gray).

In order to further study the antiproliferative activity of R2PLx, PC-3 cells were used as R2PLx showed its most potent effect on these cells. The rate of LDH release causing by R2PLx in PC-3 cells is less than 40% of maximum LDH released control (lysis buffer) at the highest concentration, which induces almost 100% of inhibition on cell viability (Figure 6A). Meanwhile, the kinetics of cytoplasmic LDH release from cancer cells were observed, whereas, at low peptide concentration ranging from 10 to 1 µM, there are no significant LDH leakage within 12 h (Figure 6C). In other words, the antiproliferation of cancer cell mechanism of action by R2PLx was not largely correlated with the disruption of plasma membrane integrity. To further investigate the mechanism of action of R2PLx, Annexin V-FITC/PI staining was used to evaluate morphological changes in treated cells (Figure 6B). After 6 h of challenge, phosphatidylserine (PS) exposure on membrane was visible in green via binding with Annexin V-FITC. Following 24 h of incubation, most cells stained by both. Annexin V- FITC and PI positive indicated that R2PLx was exerting its antiproliferative effects through a mechanism involving apoptotic processes. Furthermore, the Caspase-3 activity was also observed after 6 h of treatment with R2PLx (Figure 6D), and was found similar to Annexin V-FITC/PI staining results, as well as the low LDH leakage at 6 h (Figure 6C), suggesting R2PLx-induced cancer cell death involved in the activation of intrinsic apoptotic pathway.

## Discussion

In the present study, the R2PLx peptide was cloned from pickerel frog skin secretions and the presence of the mature peptide was confirmed by LC-MS/MS. In the various biological assays performed, R2PLx demonstrated broadspectrum antimicrobial activity, along with moderate haemolytic effects, results which are similar to the other ranatuerin-2 peptides, such as ranatuerin-2TRa, ranatuerin-2Ma or -2Mb [13,14]. So far, it is unclear how these AMPs impact upon microorganisms and cancer cells, but AMPs generally exhibit a nonspecific interaction with bacterial cell membranes, or mammalian plasma membranes, leading to the loss of integrity and ultimately disintegration of the cell [15]. Although the present study did not explore the antibacterial mechanism of R2PLx, extensive investigations have indicated that cationic AMPs possess selective toxicity towards a variety of bacteria and fungi due to the characteristics of the microbial cell membranes, such as the negative charge [15,16], which allows the cationic AMPs to aggregate on the bacterial cell surface by electrostatic attraction. By contrast, mammalian cell membranes are largely composed of cholesterol and zwitterionic phospholipids, thereby reducing their attractiveness to cationic AMPs [17].

AMPs have also shown great potential as new anticancer therapeutics [18,19], and here indeed, R2PLx can potently inhibit the proliferation of tumour cells. It is generally proposed that the antiproliferative mechanism of AMPs on cancer cells is similar to their antimicrobial action, based upon the observation that tumour cells can also possess a negatively charged membrane [20]. Indeed, here the activity of R2PLx against cancer cells is more potent than its antimicrobial activity. In addition, the observation that its IC_50_ against the cancer cells is at least 4-fold lower than against the HMEC-1 normal cells, and there is limited haemolysis at these values, indicates these peptides are selective towards the cancer cells. Also, the lack of haemolysis at the IC_50_ values for the cancer cells could indicate the mechanism of action of these peptides may not be related to membrane-lytic activity [21]. Almaaytah and co-workers also performed same dual staining method and indicated that AamAP-S1 peptide was not killing cells through apoptosis induction as they observed both AnnexinV-FITC and PI positive results [22]; however, they only displayed the results after 24 h of drug treatment. It is believed that, in the earlier events of apoptosis, translocation of membrane PS from the inner side (but integrated plasma membrane) can merely cause AnnexinV-FITC positive and subsequently, the loss of membrane integrity occur in the late period, which leads to both AnnexinV-FITC and PI positive [23–25]. In other words, although translocation of PS at the external cell surface is present in apoptosis and necrosis, the cell membrane remains intact during the initial stages of apoptosis. Herein, our observation (AnnexinV-FITC positive /PI negative in 6 h and both AnnexinV-FITC /PI positive in 24 h) was consistent with above dynamic process. Hence, we indicated that the selective cytotoxicity of R2PLx towards the cancer cells is associated with nonmembranolytic action. Moreover, we also observed the significant Caspase-3 activity in R2PLx-induced PC-3 cancer cells after 6 h of treatment, which is supposed to be the early apoptosis stage, implying R2PLx is able to trigger apoptosis cell death. In fact, previous research indicated that the change of membrane surface charge in malignant cells (the inner leaflet membrane of normal cell is relatively anionic and negative than outer) plays a key role in signalling protein (i.g., K-ras) localisation and activity, inspiring cancer cell ability to grow [17,26]. It is evident that, apart from larger negative charge on the malignant cell surface due to higher content of anionic molecules, such as O-glycosylated mucins, sialylated gangliosides and heparan sulphate, greater number of microvillus increasing surface area helps the accumulation of cationic peptides on the cancer cell membrane [27]. From these terms, it is possible for cationic R2PLx peptide to act on acid phospholipid and form lipid-peptide domain: finally such change may localise signalling protein to plasma membrane, further regaining apoptosis signalling for cancer cells and leading cell death. Also, one of the possibilities is that R2PLx might be able to bind the cell-surface death receptors (such as CD95, DR4 and DR5) as pro-apoptotic ligand, thereby inciting a cascade of intracellular signalling [28]. Alternatively, it is possible that R2PLx translocates into the cell and could be capable of interfering with intracellular organelles and/or molecules [17]. Indeed, a previous report from Cruz-Chamorro and colleagues found that magainin-1 induced apoptosis in leukaemia cells by entering the cell and causing release of cytochrome c from mitochondria [29].

We also observed that removal of the ‘rana box’ from this peptide decreased its bioactivities considerably. Among the naturally occurring AMPs from Ranidae, the ‘rana box’ structure is prevalent among the major families, which includes ranatuerin-1, brevinin-1 and -2, esculentin-1 and -2, ranalexin, vigrocin and paluserin [30]. However, the amino acid sequence of these rana box domains have been shown to consist of 6–11 residues in diverse constitutions [31]. Moreover, the importance of the rana box to the bioactivities of these peptides are variable, with the removal of this domain decreasing the antimicrobial activity of brevinin [32], but not impacting upon the function of Esculentin-1 [33]. Therefore, the study of the ‘rana box’ deleted analogue, R2PLx-22, was necessary to illustrate the influence of the rana box on the bioactivity of ranatuerin peptides. Our investigations indicated that the loss of net positive charge of R2PLx (both R2PLx-22 and S^−24^-R2PLx) demonstrates more less toxicity towards normal mammalian cells, along with less potent antibacterial and antiproliferative activities. On the other hand, the reservation of loop structure, but the absence of cationic amino acid within this domain maximum reduces the antibacterial effect, in comparison of other analogue, indicating that cationicity of rana box domain is also crucial for maintaining its biological activities. Indeed, the heptapeptide C-terminal ‘rana box’ was found to be important for maintaining both the antimicrobial and antiproliferative activity of this peptide. However, there is no agreed understanding regarding the exact role of such an intramolecular disulphide-bridge cysteine motif. Subasinghage et al. [34] used solid state nuclear magnetic resonance spectroscopy to analyse its structure in ranatuerin-2CSa, and they found the rana-box domain possesses a helix structure. Our CD spectra also proved that the removal of the rana box markedly reduced the amount of helical conformation within this peptide. Accordingly, the rana box loop of ranaturein-2 might participate in the formation of an α-helical structure in lipid membranes that correlates with antimicrobial activity and antiproliferative potency, although, this will require confirmation.

In summary, these results indicate that R2PLx is a potential candidate as an effective agent to target cancer cells given its apoptotic action and selective toxicity to human cancer cells. In addition, we have also shown that, as observed in a previous study [32], the rana box in the C-terminus of R2PLx, as well as the net positive charge, are essential for maintaining the biological potency of this peptide family.

## Supporting information

**Table S1.** Two-way ANOVA analysis of the effect on cell proliferation of R2PLx and 22the analogues on the cancer cell lines H157, PC-3, U251MG, and MCF-7 as well as HMEC-1. * p<0.05; ** p<0.01; *** p<0.001 and **** p<0.0001; ns: no significance.

## Abbrevations

AMP: antimicrobial peptide
CAP: cutaneous antimicrobial peptide
CFU: colony forming unit
CD: circular dichroism
FITC: fluorescein isothiocyanate
LDH: lactate dehydrogenase
MBC: minimum bactericidal concentration
MIC: minimal inhibitory concentration
MRSA: methicillin-resistant *Staphylococcus aureus*
OD: optical density
ORF: open reading frame
PI: propidium iodide
PS: phosphatidylserine
R2PLx: ranatuerin-2PLx
TFA: trifluoroacetic acid
TFE: trifluoroehanol
