# Supplementary material for: A novel antimicrobial peptide, Ranatuerin-2PLx, showing therapeutic potential in inhibiting proliferation of cancer cells
Source: Biosci Rep. 2018 Nov 9;38(6):BSR20180710. doi: 10.1042/BSR20180710 (PMC6239254; doi:10.1042/BSR20180710)
Supplement: Supplementary file 1 [file bsr20180710_Supp1.pdf]

A novel antimicrobial peptide, Ranatuerin-2PLx (R2PLx), showing therapeutic potential in inhibiting proliferation of cancer cells.

Xiaoling Chen[1], Luyao Zhang[1], Chengbang Ma[1], Yingqi Zhang[2,*], Xinping Xi[1,*], Lei Wang[1], Mei Zhou[1], James F. Burrows[1] and Tianbao Chen[1].

[1] School of Pharmacy, Queen's University Belfast, Belfast BT9 7BL, Northern Ireland, U.K

[2] Department of Emergency Medicine, The First Hospital of Hebei Medical University, Shijiazhuang 050031, China

*Corresponding authors: Yingqi Zhang zhangyingqi08@sina.com; Xinping Xi x.xi@qub.ac.uk

Supporting Information

Table S1. ~~One~~Two-way ANOVA analysis of the effect on cell proliferation of R2PLx and ~~R2PLx-22~~the analogues on the cancer cell lines H157, PC-3, U251MG, and MCF-7 as well as HMEC-1. * p<0.05; ** p<0.01; *** p<0.001 and **** p<0.0001; ns: no significance.

~~Tukey's multiple comparisons test~~	~~H157~~	~~PC-3~~	~~U251-MG~~	~~MCF-7~~	~~HMEC-1~~
~~Log peptide (M) = -4~~					
~~R2PLx vs. R2PLx-22~~	~~****~~	~~****~~	~~****~~	~~****~~	~~**~~
~~Log peptide (M) = -5~~					
~~R2PLx vs. R2PLx-22~~	~~****~~	~~****~~	~~ns~~	~~**~~	~~ns~~
~~Log peptide (M) = -6~~					
~~R2PLx vs. R2PLx-22~~	~~ns~~	~~ns~~	~~ns~~	~~ns~~	~~ns~~
~~Log peptide (M) = -7~~					
~~R2PLx vs. R2PLx-22~~	~~ns~~	~~ns~~	~~ns~~	~~**~~	~~ns~~
~~Log peptide (M) = -8~~					
~~R2PLx vs. R2PLx-22~~	~~ns~~	~~ns~~	~~ns~~	~~ns~~	~~ns~~
~~Log peptide (M) = -8~~					
~~R2PLx vs. R2PLx-22~~	~~ns~~	~~ns~~	~~ns~~	~~ns~~	~~ns~~

**Tukey's multiple comparisons test**	**H157**	**MBD-MB-435S**	**PC-3**	**U251-MG**	**MCF-7**	**HMEC-1**
**Log peptide (M) = -4.**						
**R2PLX vs. R2PLX-22**	****	****	****	****	***	****
**R2PLX vs. S-24-R2PLX**	*	****	****	****	****	****
**R2PLX-22 vs. S-24-R2PLX**	***	*	ns	***	*	ns
**Log peptide (M) = -5.**						
**R2PLX vs. R2PLX-22**	****	**	****	*	ns	ns
**R2PLX vs. S-24-R2PLX**	***	*	****	****	*	ns

R2PLX-22 vs. S-24-R2PLX	ns	ns	ns	***	ns	ns
Log peptide (M) = -6.						
R2PLX vs. R2PLX-22	ns	ns	ns	ns	ns	ns
R2PLX vs. S-24-R2PLX	ns	*	ns	ns	ns	ns
R2PLX-22 vs. S-24-R2PLX	ns	ns	ns	ns	ns	ns
Log peptide (M) = -7.						
R2PLX vs. R2PLX-22	ns	ns	ns	ns	ns	ns
R2PLX vs. S-24-R2PLX	ns	ns	ns	*	**	ns
R2PLX-22 vs. S-24-R2PLX	ns	ns	ns	ns	ns	**
Log peptide (M) = -8.						
R2PLX vs. R2PLX-22	ns	ns	ns	ns	ns	ns
R2PLX vs. S-24-R2PLX	*	ns	ns	*	ns	ns
R2PLX-22 vs. S-24-R2PLX	ns	ns	ns	ns	ns	ns
Log peptide (M) = -9.						
R2PLX vs. R2PLX-22	ns	ns	ns	ns	ns	ns
R2PLX vs. S-24-R2PLX	***	ns	ns	ns	ns	ns
R2PLX-22 vs. S-24-R2PLX	ns	ns	ns	ns	ns	ns
